# Does the League Table Lie? The Development and Validation of the Perceived Performance in Team Sports Questionnaire (PPTSQ)

**DOI:** 10.3389/fpsyg.2020.615018

**Published:** 2021-01-21

**Authors:** Lael Gershgoren, Asaf Blatt, Tal Sela, Gershon Tenenbaum

**Affiliations:** ^1^School of Behavioral Sciences, The College of Management Academic Studies, Rishon LeZion, Israel; ^2^Department of Behavioral Sciences, School of Social Sciences and Humanities, Kinneret Academic College on the Sea of Galilee, Emek HaYarden, Israel; ^3^Ivcher School of Psychology, Interdisciplinary Center (IDC), Herzliya, Israel; ^4^College of Education, Department of Educational Psychology and Learning Systems, Florida State University, Tallahassee, FL, United States

**Keywords:** sport psychology, perceived performance, objective outcome, team operation, effort, base-rate bias

## Abstract

Objective performance measures are vastly used in sport psychology despite their inherent limitations (e.g., unaccounted baseline differences). Founded on the nature of group goals in team sports, we aimed at developing the Perceived Performance in Team Sports Questionnaire (PPTSQ) to capture the team members’ perception of their team’s performance. Accordingly, three dimensions were hypothesized: *effort investment, skills execution*, and *perceived outcome*. To measure these dimensions, items were generated to address the players’ perception of their team performance as a whole. Four samples of athletes were used to test the psychometric properties of the PPTSQ: professional (*n* = 231), collegiate (*n* = 222), professional—retest (*n* = 89), and mixed professional–collegiate (*n* = 139). Exploratory and confirmatory factor analyses were used to estimate construct and content validities. These procedures revealed a better data fit to a two-dimensional model that consists of effort investment and perceived outcome. The reliability analyses for the PPTSQ provide satisfactory evidence that the questionnaire is a reliable measure of perceived performance in team sport. Adequate internal consistency emerged for both dimensions (0.75 < ω < 0.89). Furthermore, a high correlation was obtained for temporal stability. Concurrent validity was addressed by correlating the PPTSQ scores with the Group Environment Questionnaire and the Team Assessment Diagnostic Instrument. Correlational analysis between the PPTSQ and an objective measure of performance was used to test its predictive validity. The correlations strongly support the concurrent and predictive validities of the PPTSQ. We conclude that our perceived performance questionnaire can address various objective measures shortcomings (e.g., considering base-rate biases) resulting in a more meaningful team performance metric. Implication of the PPTSQ for sport psychology research and applied work enhancement are discussed in detail.

## Introduction

Being the end result, performance is one of the most used variables in sport psychology research. Several examples include the relationship between performance and anxiety (e.g., Craft et al., 2003), motivation (e.g., Gershgoren et al., 2011), self-efficacy (e.g., Moritz et al., 2000), and emotions (e.g., Lazarus, 2000). In team sports, the relationship between team performance and cohesion as well as Shared Mental Models (SMM) has been established both conceptually (e.g., Carron et al., 1985; Eccles and Tenenbaum, 2004, Eccles and Tenenbaum, 2007) and empirically (e.g., Gershgoren et al., 2013; Filho et al., 2014). Nevertheless, despite its empirical and methodological importance, neither a chapter on performance measures nor on perceived performance were published in the books on measurement in sport and exercise psychology of both Duda (1998) and Tenenbaum et al. (2012). This may represent a deficiency in performance measures in the sport psychology literature in general and sport psychology research in particular.

Exploring the sport domain, one can clearly identify that actual outcome measures such as win–loss percentage, points gained, and ranking are more commonly used than perceived ones. The common notion that “the league table doesn’t lie,” may be true in deterministic terms. However, “relative to expectations” terms can provide additional reliable and valuable data. A few anecdotal evidences are next presented to support the necessity of a valid and reliable perceived performance questionnaire in team sports being the purpose of this study.

In deterministic terms, a successful objective outcome, such as gaining 82 points out of 114 (i.e., 72% success), is better than gaining 56 points (i.e., 49% success; ESPN, 2020). However, subjectively, FC Barcelona considered the 2019–2020 season as a failure because the team failed to gain the championship from the rivalry team Real Madrid CF, although investing 255 million Euros in transferring players before the season of 2019–2020 (Transfermarket, 2020a). Consequently, two head coaches were fired from the team at that season, and its superstar, Lionel Messi, requested to leave the club. On the other hand, with only a 49% success rate, Granada CF ended the same season at the seventh place in the Spanish premier league as their most successful season since 1974 (Transfermarket, 2020b).

Another example comes from the first game of 2018 FIFA World Cup group stage E, where the national soccer team of Brazil, aspiring to win its sixth world cup title, tied with Switzerland who made effort to rebuild itself after the failure of disqualifying to the quarter final in the UEFA Euro 2016. At the post-game press conference, Switzerland’s head coach, Vladimir Petkovic, noted: “we were able to do it well, and it is an excellent starting position… I’m very proud and pleased with the discipline and with the way we played” (BBC Sport, 2018). In contrast, Tite, Brazil’s head coach, claimed: “there is some more work to be done.” When asked about the matches’ score, he replied, “my expectation, of course, was to get a victory, and of course, I’m not happy with the result.” Having the objective result being a tie, one may ask why the head coaches of both national teams differed as much in reviewing the result of this game.

Using an objective outcome may be appropriate assuming that all teams are similar in capabilities such as physical, technical, mental, and tactical. However, this rarely is the case in sports. Indeed, all teams start the season with no points, no win–loss percentage, and with a similar ranking. Nevertheless, teams’ capabilities are not equal causing objective outcomes to misrepresent base-rate information and thus teams’ performances expectations. Because equal baseline cannot be presumed, perceived performance must be considered aside the teams’ objective outcome and standing. Perceived performance measures are administered post-performance but may also incorporate pre-performance information such as prior expectations (i.e., was the actual outcome better or worse than expected?). The idea of unequal baseline is also reflected in sports gambling, as the winning/losing odds allocated for each team in any sports competition are rarely even. Furthermore, these odds are dynamic and are updated as the season progresses, meaning that the baseline information is complex and constantly changing. We suggest that considering inequalities among teams at baseline has meaningful consequences of how the teams’ performance is conceived, and thus must be accordingly operationalized.

To account for inequality in the team’s initial capacities, and consequently its performance expectations, one must consider “baseline rates” in order to evaluate and assess the teams’ performances. In this vein, Kahneman and Tversky (1973) laid the foundations for investigating the human tendency to neglect a prior statistical probability in favor of the most representative case. The notion of base rate bias was developed to account for judgment predictions that violate the logic of statistical likelihood (Bar-Hillel, 1980) and is reflected in peoples’ tendency to judge the likelihood of a situation without considering all relevant data. On a broader view, this notion suggests that a judgment that relies merely on “the bottom line” is fairly over simplistic and hence, may be, in cases, misrepresentative. For instance, Medvec et al. (1995) have examined the Barcelona Olympic Games and found that, despite their ranking, bronze medal winners (3rd place) expressed significantly more positive emotions than silver medal winners (2nd place) on the podium.

**FIGURE 1:**
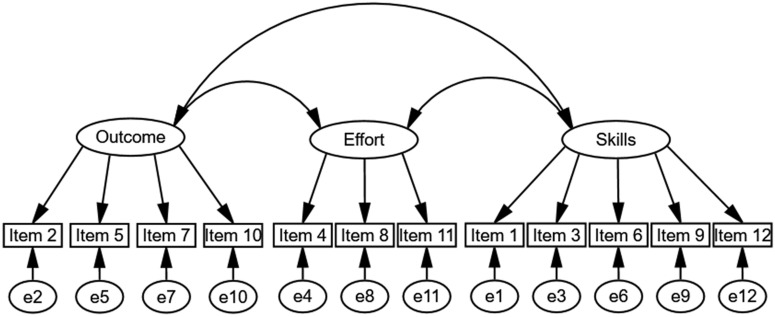
A conceptual three-factor model of the Perceived Performance in Team Sports Questionnaire (PPTSQ).

The Perceived Performance in Team Sports Questionnaire (PPTSQ), which we have developed herein, aims at capturing the team members’ perception of their team performance at any stage throughout the season. The conceptual framework guiding the development of the PPTSQ stems from Brawley et al. (1992) findings pertaining to team goal achievement in collegiate team sports. Their results revealed that goals for competitions were effort, skill, or outcome related. Accordingly, the PPTSQ was designed to capture the team members’ perception of three factors: *effort investment*, *skills execution*, and *perceived outcome*. The conceptual three-factor model of the PPTSQ is presented in Figure 1. To measure these factors, items were generated to address the players’ perception of their team performance as a whole. This approach was supported by Feltz et al. (2008) claiming that team aspects can rightfully be measured through each member’s appraisal of the team.

Because objective measures of performance may overlook fundamental differences among teams, only a moderate positive correlation was expected among the PPTSQ dimensions and the objective performance measure. Consisting of the conceptual relationship between cohesion and performance (e.g., Widmeyer et al., 1985) and the overall effect sizes reported by the meta-analysis of Filho et al. (2014, *ES* = 0.34), a moderate and positive correlation was anticipated between the PPTSQ and the cohesion measure being used. Furthermore, stemming from Eccles and Tenenbaum’s (2004, 2007) conceptual framework of shared cognitions in expert teams and the correlation (*r* = 0.36) reported by Webber et al. (2000) between SMM and performance, a positive and moderate correlation was expected between the PPTSQ and the SMM questionnaire being administered to team members.

## Materials and Methods

### Participants

Four samples were used to estimate the statistical properties of the PPTSQ: professional athletes (*n* = 231), collegiate athletes (*n* = 222), professional athletes—retest (*n* = 89), and mixed professional–collegiate athletes (*n* = 139). Demographic data of these samples are presented in Table 1. The first sample contained both male and female (*n* = 154 and 77, respectively) professional athletes from 25 teams with an average of 9.2 athletes per team (*SD* = 3.7). The second sample, collegiate athletes, was composed of both genders (126 male athletes and 96 female athletes) participating in 32 teams (*M* = 6.9 athletes per team, *SD* = 3.7). Third, 89 professional male and female athletes (*n* = 48 and 41, respectively) from the first sample completed a retest of the PPTSQ. This sample was explored in relation to their first administration (i.e., temporal stability) and independently (i.e., as an additional confirmatory analysis). The last sample, mixed professional (*n* = 62) and collegiate (*n* = 77) athletes, also included both male and female athletes (*n* = 83 and 56, respectively) from 39 teams with an average of 3.6 athletes per team (*SD* = 2.8). This sample was used only to reconfirm the structural model and was composed of participants with missing data from samples 1 and 2. We refrained from using these participants in the original exploratory factor analysis (EFA) and confirmatory factor analysis (CFA) analyses to perform a full-case analysis to establish and validate our structural model.

**TABLE 1:** Samples and demographic data.

**Sample**	**Sample type**	***N***	**Gender (Male/Female)**	**Age (*SD*)**
Sample 1	Pro	231	154/77	24.61 (4.80)
Sample 2	Col	222	126/96	24.95 (2.97)
Sample 3—retest	Pro	89	48/41	24.82 (3.03)
Sample 4—mixed	Pro + Col	139	83/56	23.80 (3.59)

*Pro, Professional level; Col, College level.*

Four criteria and justifications were employed for participation in this study. First, participants were active members in professional or collegiate level teams from various sports such as soccer, basketball, futsal, volleyball, rugby, water polo, and team handball. Second, all the athletes were 18 years of age and above. Team strategies and tactics are mostly trained and best acquired at least at the late stages of adolescence (i.e., collegiate and professional levels). Third, to provide meaningful data pertaining to team performance, team members were required to share three or more competitive experiences (i.e., played at least three games together). Last, in accordance with the institutional review board (IRB) committee guidelines, participants were, regardless of their original nationalities, active members of teams that competed in Israeli leagues/tournaments.

### Instrumentation

Three questionnaires were administered in this study. The PPTSQ was administered to examine the members’ perceptions pertaining to various performance-related components. In addition, team cohesion and SMM questionnaires were administered to validate the PPTSQ. Furthermore, objective data regarding the team’s outcome scores were collected. To capture the sample’s characteristics, a demographic form was administered.

Since this study took place in Israel, in which Hebrew is the native spoken language, a back-translation procedure was conducted. Hence, all instruments were translated to Hebrew and then back-translated to English. No noteworthy misfits were observed. These translations were made by two separate individuals who were very familiar with both the Hebrew and the English languages. A back-translation procedure is commonly used in such instances and is supported in the extant literature (see Brislin, 1986). Eventually, both the English and the Hebrew versions were available to the athletes and were provided according to their request.

#### Perceived Performance in Team Sports Questionnaire

The development and validation of the PPTSQ followed a commonly used procedure (e.g., Chatterji et al., 2002; Johnson et al., 2007) and consisted of six stages.

Items Generation. This stage involved a thorough literature review pertaining to performance measures in sport. In addition, an examination of team-related goals afforded the emergence of a performance conceptual framework that was used to generate 12 items under three factors pertaining to performance perception in team sports. Accordingly, the *effort investment* scale included items 4, 8, and 11; the *skills execution* scale contained items 1, 3, 6, 9, and 12; and the *outcome* scale included items 2, 5, 7, and 10. Items were scored on a 5-point bipolar Likert scale where each item contained two unique contradicting/opposite statements at the continuum ends and a statement at its mid-point. All the items of the original PPTSQ are presented in Supplementary Appendix A.

Content Validity. New measures must adequately capture the variable under examination eliminating superfluous content (Hinkin, 1995). Hence, the items’ *relevance* and *representativeness* to the domain of interest are at the forefront of content validity assessment (Vaughn and Daniel, 2012). This judgment procedure is usually performed by experts in the domain of interest. In the current study, content/face validity of the PPTSQ was obtained by two respected scholars in the sport psychology domain in general and in team processes in particular.

Descriptive Statistics. Descriptive statistics aims at calculating central tendencies and distribution variables (i.e., mean, *SD*, skewness, and kurtosis) of each item, items within factors, and the entire questionnaire. This stage was implemented following data collection.

Construct Validity. Construct validity is aimed at accounting for as much as possible the distinctive variance of each of the variables while grouping them into clusters. Because the PPTSQ does not rely on an *a priori* model but only on a conceptual framework, the model fit to the data was examined through an EFA procedure followed by several confirmatory procedures.

Reliability. Reliability (i.e., internal consistency) was obtained using McDonald’s omega (ω) for each scale. Recently, Hayes and Coutts (2020) have presented McDonald’s omega (ω; see also McDonald, 1999; Hancock and An, 2020) as a superior reliability measure to the traditional Cronbach’s alpha (α). Temporal stability was also estimated, as the PPTSQ was readministered to a subset sample of 89 professional athletes with an average of 8 days apart between the two administrations.

Concurrent and Predictive Validity. Concurrent validity was addressed by correlating the PPTSQ scores with the Group Environment Questionnaire (GEQ; Carron et al., 1985). Furthermore, concurrent validity was also tested by correlating the PPTSQ with the Team Assessment Diagnostic Instrument (TADI; Johnson et al., 2007), which is an SMM questionnaire. Correlational analysis between the PPTSQ and an objective measure of performance was used to test its predictive validity.

#### The Group Environment Questionnaire

The GEQ (Carron et al., 1985) was designed to measure group cohesiveness. The conceptual framework, which guided the development of the GEQ, was derived from the notions of task and social cohesions stemming from individual and team level perspectives (see Widmeyer et al., 1985). Social cohesion pertains to the degree to which group members are bonded or close to one another while interacting socially. Task cohesion refers to the degree to which group members remain united while striving to complete their task or achieve their performance-related goals (see Festinger et al., 1963; Carron, 1984).

Four subscales are used to capture team cohesion: *interpersonal attraction—social (ATGS)*; *interpersonal attraction—task (ATG-T)*; *group integration—social (GI-S)*; and *group integration—task (GI-T)*. The interpersonal attraction subscale consists of nine items (i.e., five social cohesion items and four task cohesion items). The group integration subscale also includes nine items (i.e., four social and five task cohesion items). All items are scored on a 9-point Likert-type scale ranging from *strongly disagree* (1) to *strongly agree* (9). Twelve items (i.e., 1, 2, 3, 4, 6, 7, 8, 11, 13, 14, 17, and 18) are negatively worded and reversed in scoring.

Cronbach alpha coefficients for the ATG-S, ATG-T, GI-S, and GI-T were found to be *r* = 0.64, 0.75, 0.76, and 0.70, respectively (Carron et al., 1985). Using CFA, Li and Harmer (1996) have confirmed the four-factor model of the GEQ as initially suggested by Carron et al. Criterion validity of the GEQ was obtained by identifying a moderate correlation with the team perception scale of the Sport Cohesiveness Questionnaire (Martens et al., 1971) and moderate correlations between the task subscales in team sports and the roles related items of the Team Climate Questionnaire (Grand and Carron, 1982). As predicted, a non-significant correlation was obtained between the GEQ and the Bass Orientation Inventory (Bass, 1962), suggesting that one’s personal motivation is unrelated to one’s appraisal of his/her team’s cohesion. In conclusion, the GEQ is by far the most widely used cohesion questionnaire in sport (Carron et al., 2002).

#### Team Assessment Diagnostic Instrument

The TADI (Johnson et al., 2007) is a measure of team SMM content. Five team processes (i.e., SMM factors) emerged from the EFA analysis accounting for 82% of the variance: *General Task and Team Knowledge* (GTTK), *Communication Skills (CS)*, *Attitude Toward Teammates and Task* (ATTT), *Team Dynamics and Interactions* (TDI), and *Team Resources and Working Environment* (TRWE). The Cronbach’s alpha coefficients of the SMM factors (i.e., 0.76, 0.89, 0.75, 0.81, 0.85, respectively) suggest adequate reliability (i.e., internal consistency). Furthermore, at a later stage of their study, Johnson et al. have verified the five-structure model through a CFA procedure.

The TADI includes 15 items in total, 3 for each team process, rated on a 5-point Likert scale. Items response is anchored by *strongly disagree* (1) and *strongly agree* (5). A team score is calculated by averaging all the items’ scores for each team member followed by averaging all the team members’ mean scores. This score was interpreted as the perception of the team members pertaining to their overall level of SMM. Higher mean score indicates higher levels of sharedness. Similar mathematical reasoning was used for each team process independently. Such calculation afforded capturing the sharedness level of each of the SMM factors separately.

#### A Standardized Objective Performance Score

Because our teams performed in different types of competitions (i.e., league vs. tournament) and under different scoring systems (e.g., soccer where a win grants 3 points and a loss grants 0 vs. basketball where a win grants 2 points and a loss grants 1 point), the performance scores were standardized. Thus, the objective outcome score was calculated as the percentage of points earned from the maximum possible points when a loss, a tie, and a win grant 0, 1, and 2 points, respectively. For example, if a futsal team won three games, tied two, and lost two, a total of 8 points was considered. This value was transferred to a percentage value resulting in a 57.1% (8 points earned divided by 14 possible multiplied by 100) success rate. Success rate values were calculated only for the official records of the professional leagues/tournaments.

### Procedure

All teams completed the questionnaire at the end of a tournament or the end of their regular season. At first, permission to approach the athletes was obtained from a team representative (e.g., athletic director, head coach, team’s owner). Then, a convenient time for administration was coordinated with the head coach either at the tournament venue or at the team’s site. When repeated measures took place, both dates were scheduled in advance with approximately 1 week apart. Following the IRB-approved protocol, informed consents were obtained from the athletes, and confidentiality was verbally announced prior to data collection. Specifically, participants were informed that only the researchers will have access to the players’ responses. The athletes were asked to complete the demographics form first followed by a battery of questionnaires including the PPTSQ, GEQ, and the TADI. A counter-balanced order was used to eliminate order effects due to fatigue or loss of interest, which can negatively affect the response quality and consequently increase the measurement error. The retest administration included only the PPTSQ. Prior to the retest, confidentiality was announced again. At the end of each administration, once the athletes completed the questionnaire, they were thanked and released.

### Statistical Analysis

The analytic approach consisted of several stages. First, using sample 1 (professional athletes), we fitted different exploratory models. We started with a three-factor model based on our conceptual framework that included 12 items composing three latent factors: effort investment, skills execution, and perceived outcome. We evaluated this model alongside other models using Kline’s (2015) fit indices: Standardized Root Mean square Residual (SRMR < 0.08), Adjusted Goodness of Fit (AGFI ≥ 0.90), Comparative Fit Index (CFI ≥ 0.90), Root Mean Square Error of Approximation (RMSEA < 0.08), and Bayes Information Criterion (BIC), to establish the best-fitted model. Second, after establishing the best-fitted model, we reexamined this model by a CFA using three additional samples (college-athletes sample, professional athletes retest sample, and mixed sample). Next, we used bivariate correlations to estimate the concurrent validity between PPTSQ factors and TADI and GEQ scores. Finally, the predictive validity of the PPTSQ was obtained by correlating its factors’ scores with the scores of the standardized objective performance measure. Reliability (i.e., internal consistency) was obtained using McDonald’s omega (ω; Hayes and Coutts, 2020). Omega estimates reliability more accurately than Cronbach’s alpha when the tau-equivalence assumption (equal factor loadings of all items) is violated (as frequently is the case; Trizano-Hermosilla and Alvarado, 2016). Furthermore, Cronbach’s alpha can be high even if a set of items measures more than one construct because it only *assumes* a construct’s unidimensionality (Green et al., 1977; Graham, 2006) but fails in measuring it directly as such. Omega, on the other hand, overcomes these limitations and does not rely on the tau-equivalence assumption and consist of a single-factor solution extracted from a factor analysis (see Hancock and An, 2020; Hayes and Coutts, 2020). Temporal stability was estimated via Pearson correlation. Data were analyzed using IBM SPSS Statistics for Windows, Version 25.0 (IBM Corp., Armonk, NY) and IBM SPSS Amos Version 24.

## Results

The development and validation of the PPTSQ followed a six-stage procedure. These stages included item generation, content validity, descriptive statistics, construct validity, reliability, and concurrent and predictive validity. The first two stages are related to the development of the PPTSQ and were conducted before data collection. These stages were elaborated on in the method section and, hence, will not be repeated in this section.

### Descriptive Statistics

Prior to testing the study’s hypotheses, central tendencies and distribution statistics (i.e., mean, *SD*, skewness, and kurtosis) of all the PPTSQ items and scales were examined in the first sample (professional level; *N* = 231; see Table 2). Items’ mean ranged from 2.81 (item 10) to 3.74 (item 8); *SD* ranged from 0.98 (item 12) to 1.27 (item 2). Skewness and kurtosis values for all the items ranged between –0.94 and 0.03, suggesting no substantial deviations from normality in the item-response distributions.

**TABLE 2:** Descriptive statistics of the original PPTSQ total score, scales, and items.

**Scale**	**Mean**	****SD****	**Skewness**	**Kurtosis**
Total score	3.21	0.86	–0.32	–0.36
Effort investment	3.18	0.91	–0.26	–0.44
Skills execution	2.94	1.06	–0.19	–0.71
Perceived outcome	3.61	0.91	–0.52	0.15
Item 1	3.39	1.10	–0.50	–0.24
Item 2	2.90	1.27	0.00	–0.94
Item 3	3.16	1.16	–0.36	–0.50
Item 4	3.43	1.08	–0.41	–0.13
Item 5	3.19	1.19	–0.35	–0.56
Item 6	2.97	1.15	–0.18	–0.74
Item 7	2.87	1.15	–0.08	–0.67
Item 8	3.74	1.06	–0.59	–0.12
Item 9	3.10	0.99	–0.28	–0.19
Item 10	2.81	1.18	0.03	–0.72
Item 11	3.64	1.10	–0.55	–0.23
Item 12	3.26	0.98	–0.27	–0.03

### Construct Validly (Convergent and Discriminant Validity)

Several EFA models were tested to identify the best fit to the data (see Table 3 models 1–3). First, we fitted a three-factor model based on theoretical foundation that included 12 items composing three latent factors: effort investment, skills execution, and perceived outcome. As depicted in Table 3, this model failed to share an adequate fit to the data. Furthermore, the correlations between skills execution and the other two factors were very high (0.91 with the perceived outcome factor and 0.77 with effort investment). Thus, a two-factor model that included only the perceived outcome and effort investment factors (see Table 3, model 2) was tested. This model demonstrated a good fit to the data. Nonetheless, the omission of one item (item 5) enhanced model fit indices (see Table 3, model 3). This model was, then, determined as the best-fitted model to the data.

**TABLE 3:** Exploratory factor analysis (EFA) and confirmatory factor analysis (CFA) structural equation modeling (SEM) models for the Perceived Performance in Team Sports Questionnaire (PPTSQ)—attached in a separate file.

	**Model**	**Items**	**Sample**	***N***	**Analysis type**	**Chi-square (CMIN)**	**DF**	**Probability level**	**CMIN/DF**	**RMR**	**SRMR**	**GFI**	**AGFI**	**CFI**	**RMSEA**	**BIC**
1	Model 1: 3-factor model	12	Pro, Sample 1	231	EFA	165.562	51	p < 0.001	3.246	0.056	0.047	0.891	0.833	0.943	0.099	312.507
2	Model 2: 2-factor model	7	Pro, Sample 1	231	EFA	30.391	13	*p* < 0.005	2.338	0.042	0.0329	0.965	0.925	0.981	0.076	112.027
3	Model 3: 2-factor model*	6	Pro, Sample 1	231	EFA	15.482	8	*p* = 0.05	1.935	0.028	0.022	0.979	0.944	0.989	0.064	86.234
4	Model 3	6	Col, Sample 2	222	CFA 1	14.578	8	*p* = 0.068	1.822	0.024	0.0248	0.979	0.946	0.987	0.061	84.813
5	Model 3	6	Pro, Re-test, Sample 3	89	CFA 2	13.32	8	*p* = 0.101	1.665	0.048	0.056	0.957	0.887	0.98	0.087	71.672
6	Model 3	6	Pro + Col, Sample 4	139	CFA 3	10.575	9	*p* = 0.306	1.175	NA				0.99	0.036	NA

EFA and CFA SEM Models for the PPTSQ.*Best EFA fitted model; SRMR, Standardized root mean square residual; AGFI, Adjusted Goodness of Fit; CFI, Comparative Fit Index; RMSEA, Root Mean Square Error of Approximation; BIC, Bayes Information Criterion. Pro, Professional level, Col, College level.

**FIGURE 2:**
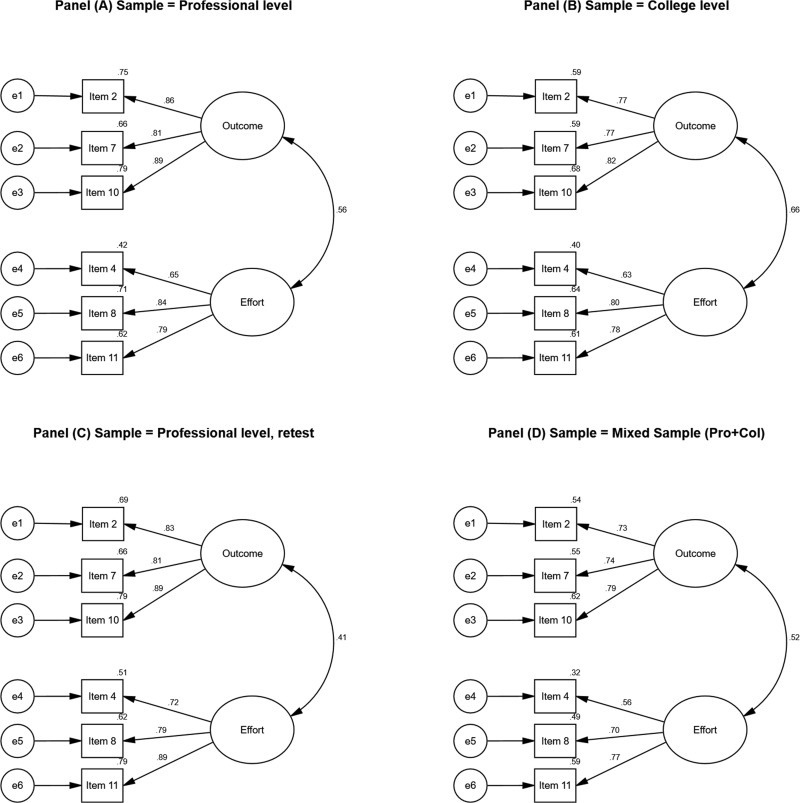
Best fitted model (Model #3 - 2-factor model) for samples 1–4. Standardized estimates are presented alongside squared multiple correlation for each item (upper left corner). All estimates are significant at the *p* < 0.001. **Panel (A)** presents results from the EFA analysis (Professional sample, *N* = 231); **Panel (B)**, presents results from the CFA analysis (College sample, *N* = 222). **Panel (C)** presents results from the CFA analysis (Professional retest sample, *N* = 89); **Panel (D)**, presents results from the CFA analysis (mixed sample, *N* = 139).

Figure 2A presents the EFA model, which includes two factors, effort investment and perceived outcome. Furthermore, the structural model was then confirmed using three additional samples. Table 3 and Figure 2B present the CFA of a two-factor model based on sample 2—the college sample. This model demonstrated a very good fit to the data. Lastly, the two-factor model was confirmed by two separate samples: (a) the professional athletes retest sample and (b) the mixed professional–collegiate sample (see Table 3 and Figures 2C,D, respectively). Table 4 presents the descriptive statistics of the PPTSQ total score and scales for models 1–4. Means ranged from 2.86 (perceived outcome, model 1) to 3.61 (effort investment, model 1); *SD* ranged from 0.66 (total score, model 4) to 1.09 (perceived outcome, model 1). Skewness and kurtosis coefficients for the total score and the scales ranged between −0.70 and 1.1. Thus, the assumption of distribution normality was conformed.

**TABLE 4:** Descriptive statistics of the modified Perceived Performance in Team Sports Questionnaire (PPTSQ) scales and total scores.

**Sample #**	**Scale**	**Mean**	***SD***	**Skewness**	**Kurtosis**
1	Effort investment	3.61	0.91	–0.52	0.15
	Perceived outcome	2.86	1.09	–0.08	–0.70
	Total score	3.23	0.86	–0.27	–0.14
2	Effort investment	3.47	0.85	–0.13	–0.24
	Perceived outcome	2.98	0.87	0.20	0.11
	Total score	3.23	0.75	0.07	–0.04
3	Effort investment	3.46	0.82	–0.54	0.93
	Perceived outcome	2.92	0.78	–0.10	0.58
	Total score	3.19	0.66	0.07	1.10
4	Effort investment	3.48	0.74	–0.18	0.36
	Perceived outcome	2.88	0.77	–0.36	0.13
	Total score	3.18	0.63	–0.15	0.13

Considering the entire study’s sample (363 male and 229 female athletes), we examined possible gender differences for mean PPTSQ scores for both PPTSQ dimensions using independent samples *t*-tests. Non-significant gender difference emerged for male and female athletes in effort investment scores (*M* = 3.54, *SD* = 0.89 vs. *M* = 3.50, *SD* = 0.80, respectively), *t*(590) = 0.553, *p* = 0.58, two-tailed. Moreover, non-significant gender difference was obtained for perceived outcome (*M* = 2.89, *SD* = 1.00 vs. *M* = 2.94, *SD* = 0.84, respectively), *t*(543.565) = –0.622, *p* = 0.53, two-tailed. Finally, non-significant gender effect for PPTSQ total score was revealed (*M* = 3.22, *SD* = 0.81 vs. *M* = 3.22, *SD* = 0.70, respectively), *t*(537.554) = –0.60, *p* = 0.95.

### Reliability

Table 5 presents the McDonald’s omega coefficients for effort investment and perceived outcome. Omega coefficients were adequate across all the samples ranging from 0.75 to 0.84 for effort investment and from 0.78 to 0.89 for perceived outcome. In addition, temporal stability for both factors was good (effort investment dimension: *r* = 0.85, *p* < 0.001; perceived outcome dimension: *r* = 0.80, *p* < 0.001). Item-scale correlations were very high (ranged from 0.79 to 0.92), demonstrating a very strong item-scale relationship. Inter-item correlations ranged from 0.42 to 0.77, suggesting both inter relationship and differentiation among the items within the scales. Item-scale and inter-item correlations for each scale are separately presented in Tables 6, 7. Considering the entire study’s sample (*N* = 592; samples 1, 2, and 4), items composing each factor were highly correlated with the total factor score ranging from 0.8 to 0.9 and only moderately related to the total score of the other factor, with correlations ranging from 0.35 to 0.46.

**TABLE 5:** McDonald’s omega coefficient (ω) for the Perceived Performance in Team Sports Questionnaire (PPTSQ) scales in the four samples.

	**Sample**			
**Scale**	**1**	**2**	**3**	**4**
Effort investment	0.81	0.78	0.84	0.75
Perceived outcome	0.89	0.83	0.88	0.78

**TABLE 6:** Item-scale and inter-item correlation matrix for the effort investment scale.

**Sample**		**Item 4**	**Item 8**	**Item 11**
1	Item 4	1		
	Item 8	0.539**	1	
	Item 11	0.507**	0.671**	1
	EI total score	0.806**	0.869**	0.861**
2	Item 4	1		
	Item 8	0.497**	1	
	Item 11	0.490**	0.628**	1
	EI total score	0.809**	0.845**	0.842**
3	Item 4	1		
	Item 8	0.523**	1	
	Item 11	0.651**	0.707**	1
	EI total score	0.850**	0.842**	0.907**
4	Item 4	1		
	Item 8	0.417**	1	
	Item 11	0.475**	0.581**	1
	EI total score	0.788**	0.797**	0.851**

**p < 0.001.EI, effort investment.

**TABLE 7:** Item-scale and inter-item correlation matrix for the perceived outcome scale.

**Sample**		**Item 2**	**Item 7**	**Item 10**
1	Item 2	1		
	Item 7	0.697**	1	
	Item 10	0.769**	0.723**	1
	PO total score	0.914**	0.886**	0.917**
2	Item 2	1		
	Item 7	0.610**	1	
	Item 10	0.628**	0.623**	1
	PO total score	0.872**	0.846**	0.874**
3	Item 2	1		
	Item 7	0.685**	1	
	Item 10	0.729**	0.727**	1
	PO total score	0.909**	0.881**	0.907**
4	Item 2	1		
	Item 7	0.513**	1	
	Item 10	0.565**	0.544**	1
	PO total score	0.833**	0.816**	0.849**

**p < 0.001. PO, perceived outcome.

### Concurrent and Predictive Validity

The correlations presented in Table 8 indicate that the PPTSQ and its two dimensions correlated significantly with TADI and GEQ scores. The PPTSQ total score was moderately correlated with the team cognitive measure (i.e., TADI; *r* = 0.56). Moderate correlative pattern was evident between the PPTSQ total score and all the TADI scales (GTTK, *r* = 0.47; CS, *r* = 0.46, ATTT, *r* = 0.48; TDI, *r* = 0.46; TRWE, *r* = 0.42). A low, yet significant, correlation was revealed between the PPTSQ and the team social construct of cohesion (i.e., GEQ; *r* = 0.24). Within the GEQ, a higher correlation was found between the PPTSQ and the GEQ group-integration scales (*r* = 0.36 with both the task and social scales) than with the attraction-to-group scales (task, *r* = 0.12; social, *r* = 0.16). Separately, the effort investment dimension moderately correlated with the TADI (*r* = 0.58), while the perceived outcome dimension demonstrated a slightly lower, yet still moderate, correlation (*r* = 0.44) with it. Similarly, the effort investment dimension shared a higher correlation with the GEQ then the Perceived outcome factor (*r* = 0.27 and 0.17, respectively). These findings support the concurrent validity of the PPTSQ measure and its dimensions. Interestingly, using a partial correlation analysis, we found that, when controlling for the perceived outcome scores, the correlation between TADI scores and objective performance diminished (*r* = 0.02, *p* = 0.80), as well as the correlation between GEQ and objective performance (*r* = –0.003, *p* = 0.97).

**TABLE 8:** Pearson correlations between Perceived Performance in Team Sports Questionnaire (PPTSQ), Group Environment Questionnaire (GEQ), Team Assessment Diagnostic Instrument (TADI), and Objective Performance for samples 1 and 2 (combined; *N* = 453).

	**(1)**	**(2)**	**(3)**	**(4)**	**(5)**	**(6)**
(1) Mean PPTSQ	1					
(2) Effort investment	0.914**	1				
(3) Perceived outcome	0.918**	0.678**	1			
(4) Mean GEQ	0.240**	0.271**	0.170**	1		
(5) Mean TADI	0.556*	0.585**	0.436**	0.288**	1	
(6) Objective performance^	0.467*	0.310**	0.527**	0.174*	0.258**	1

^N = 180 for correlation between objective performance and all other variables.*p < 0.05, **p < 0.001.

To test its predictive validity, the PPTSQ was correlated with an objective performance score. A moderate correlation (*r* = 0.47) was identified between these measures. Moreover, a moderate correlation (*r* = 0.53) was obtained between the perceived and the objective outcome scores. Such a correlation suggests that these variables are related yet distinct. Finally, effort investment correlated low-moderately (*r* = 0.31) with the objective outcome score. Noteworthy, the PPTSQ has demonstrated stronger relationships with the SMM measure (the TADI; *r* = 0.56) than the objective performance measure (*r* = 0.26). These results were also evident with the cohesion measure. The PPTSQ shared a higher correlation with the GEQ (*r* = 0.24) than the objective measure (*r* = 0.17).

## Discussion

It is often assumed that athletes’ perceived performance is equivalent to the objective outcome. However, psychological mechanisms of information processing are involved in players’ appraisals of their teams’ operations (e.g., Micklewright et al., 2009). These mechanisms are idiosyncratic and consist of relative analyses of information that are abstract in nature such as perceived effort (e.g., did the players exert sufficient amount of effort?) or absolute such as the score (e.g., are the players happy with this score considering the opponent’s skill level or how has the game unfolded?). Yet, only a few scientific studies have addressed this enigmatic notion.

In the current study, we challenged this traditional belief, where the objective performance score merely determines perceived performance in team sports. This study centered on the development and validation of the PPTSQ, a self-report measure that assesses team sports members’ perceptions about previous team performances. The PPTSQ development consisted of a phenomenological analysis that considered the nature of team goals in sports teams (Brawley et al., 1992) and by each player’s perceptions of his/her team performance as a whole (Feltz et al., 2008).

Specifically, the EFA of the PPTSQ retained six items (the final version of the PPTSQ and its six items are presented in Supplementary Appendix B) and revealed a two-factor structure: *effort investment* and *perceived outcome*. The factor of skills execution was omitted due to its very high correlation with the perceived outcome factor. Confirmatory factor analyses of three samples confirmed the PPTSQ’s EFA factor structure obtained by several fit indices (e.g., AGFI, SRMR; Kline, 2015). Importantly, the structure of the PPTSQ was confirmed on different samples of professional and collegiate athletes, supporting the generalizability (i.e., external validity) of the final structural dimensions of the measure.

Overall, the reliability analyses of the PPTSQ provide satisfactory evidence that the questionnaire is a reliable measure of perceived performance in team sport. Both internal consistency and temporal stability were examined to support the PPTSQ reliability. High internal consistency emerged for both factors of perceived effort investment and perceived outcome (0.75 < ω < 0.89^1^). Furthermore, a high correlation was obtained for temporal stability, with an average of 8 days between administrations. The temporal stability coefficients of both the perceived effort investment scale and the perceived outcome scale were deemed sufficient exceeding 0.80 (see Vaughn and Daniel, 2012).

Finally, the PPTSQ and its dimensions, perceived effort investment and perceived outcome, correlated positively and significantly with the TADI (Johnson et al., 2007) and the GEQ (Carron et al., 1985) scores. Specifically, as hypothesized, the PPTSQ and its two dimensions correlated moderately or higher with TADI. Significant and positive association, although slightly lower than hypothesized, emerged between the PPTSQ total score and its dimensions and GEQ.

The overall results of these correlational analyses support the concurrent validity of the PPTSQ. In addition, the PPTSQ was correlated with the objective measure of performance to support its predictive validity. Because the objective measure of performance was assumed to overlook fundamental differences on the one hand and play an important role on the other, a moderate correlation was expected between the introspective and objective performance measures. Indeed, the objective measure of performance demonstrated a moderate correlation with the PPTSQ total score. Yet, a slightly lower correlation was identified for the effort investment dimension, and a slightly higher correlation was obtained for the perceived outcome dimension. Overall, these findings support the predictive validity of the PPTSQ and, at the same time, emphasize its unique contribution to performance measurement.

### Theoretical Considerations

Our study pertained to how athletes perceive the performance of their teams. The “base rate” of the team (e.g., team capacities) plays a major role in the establishment of performance expectations; yet, in performance evaluation, this factor was overlooked. Herein, Base Rate Fallacy is a human tendency to neglect the base rates of a case in favor of the event-specific information (Bar-Hillel, 1980). For instance, million people play the lottery despite the scant odds against winning the jackpot. In a similar vein, in sport, one can assume that a draw in a game represents an equivalent performance satisfaction for both contenders. However, such performance satisfaction is often determined by a stochastic concept rather than a deterministic one. Stochastic analyses consider several variables (e.g., squad, budget, expectations, how the game unfolded, etc.) and, although cognitively complex, are performed by athletes and coaches almost automatically. This, almost automatic, analysis pattern of relative, multidimensional and complex information is familiar to athletes, as it characterizes their on-field decision-making processes (Tenenbaum, 2003). The controversy of judging sport’s outcome calls for theoretical principles and empirical data that concentrate on the mechanisms that account for this phenomenon. Therefore, the PPTSQ was proposed to examine team players’ appraisals following actual competitions. Overall, the current study was aimed at establishing a perceived performance questionnaire in sport and examining its psychometric properties within adequate samples.

Following the EFA procedure, the modified PPTSQ contained two scales, *perceived outcome* and *effort investment*. The former factor incorporates the expectations and the objectives of the athletes concerning team performance. Consistent with attribution theory (Weiner, 1974), these aspects are more related to one’s appraisal of ability and task difficulty and, thus, are relatively external and only partially under one’s control. Yet, they are vital to perceived performance, as they encompass the athlete’s initial expectations and appraisals of fundamental differences. The second dimension captures effort investment properties, such as commitment and intensity. These properties are relatively internal and under the player’s control (Weiner, 1974) although they can also be compared to others. Obviously, these two factors, although distinct, are interrelated. Team members who met or exceeded their performance expectations reported being highly focused and committed; vice versa, falling short of team objectives resulted in a lower perceived effort (Gould et al., 1999). Indeed, the results of the current study revealed a strong correlation (*r* = 0.68) between the PPTSQ components. However, such a correlation, although high, suggests that each dimension contributes uniquely and sufficiently to a complete understanding of the athlete’s perception of his/her performance. This conclusion is further supported by the different correlation each dimension shared with the GEQ, TADI, and objective performance measures.

Pertaining to the deletion of the *skills execution* dimension, the results indicated an extremely high correlation (*r* > 0.90) between skill execution and perceived outcome. It appears that athletes already consider skill abilities in their initial expectations and, hence, assimilate them into their appraisal of the outcome. Further work is required to verify if these two dimensions are confounded or if there are some conditions in which it is possible to dissociate them.

Interestingly, this study revealed that the PPTSQ is tied more closely to the TADI than to the GEQ. This finding indicates that perceived performance is more associated with SMM, the cognitive aspect of team performance, rather than with cohesion that, as aforementioned, is inherently a social oriented psychological construct. SMM relates to collective cognitive schemas that dynamically govern team coordination through team-related decision-making processes. Performance wise, these processes enable synchronization among the players’ actions under specific task demands (Cannon-Bowers et al., 1993; Tenenbaum and Gershgoren, 2014).

Concerning predictive validity, both the TADI and the GEQ demonstrated stronger correlations with the introspective measure of performance than the objective outcome. Since team coordination (e.g., superior SMM) and cohesion underly team performance (Gershgoren et al., 2013; Filho et al., 2014), our new measure of performance may be considered a more suitable indicator of team performance. Furthermore, when controlling for the variable *perceived performance*, the statistical relationship between the objective measure and either the TADI or the GEQ diminished. Altogether, these findings suggest that the two performance measures, objective and introspective, are confounded.

Akin with our postulations, outcome result must be addressed as context dependent (e.g., ranking); otherwise, it may be misleading. To exemplify, assume that a cohesive underdog team ties a superior non-cohesive team. The high perceived performance score of the underdog team and the low perceived performance score of the superior team will provide a correlative support to the cohesion-performance linkage that otherwise could have been remained unnoticed.

When addressing performance measurement, one must distinguish between expected and unexpected competitive outcomes. In the case of expected results (for instance, a high-ranked team dominates the scoreboard against a low-ranked team or when two relatively equal-ranked teams tie), both objective and introspective measures are reliable indicators of performance level. However, under unexpected results (e.g., when a low-ranked team ties or beats a high-ranked team, or when one team dominates a relatively equal-ranked team), only the introspective measure represents reliably the performance level of both teams. That is, the introspective measure of perceived performance remains reliable under both expected and unexpected conditions. This pattern suggests that the objective performance measure is inherent within the broader concept of perceived performance.

We believe that the PPTSQ provides an additional important contribution in facilitating group metacognition (Hinsz, 2004). Teammates engage in post-process coordination that comprises metacognition behaviors pertaining to the team performance. These include, among others, verbal discussions and video analyses (Eccles and Tenenbaum, 2004). Post-event reflection (Chow and Luzzeri, 2019) is a post-competition evaluation procedure that aims to enhance self-monitoring, self-correction, and emotional regulation skills among team athletes. Indeed, teams that engaged in task execution monitoring followed by future strategies establishment demonstrated improved performance compared to teams who did not participated in such processes (Rasker et al., 2000).

In a sense, the PPTSQ requires the subject to perform a metacognitive evaluation process on a team level. The athlete is required to reflect on how the team performed during competitions in terms of effort and whether the outcome was in line with prior expectations. Metacognition refers to a person’s knowledge of his cognitive system (Flavell, 1979). It involves reflective thinking in which the thought process itself becomes the object of observation. Different theoretical accounts put forward two main metacognitive processes: *monitoring* and *control.* Monitoring involves a subjective assessment of the quality of task performance, while control is the decision made following monitoring (Nelson and Narens, 1990). We suggest that the two dimensions retained from the PPTSQ, effort investment and perceived outcome, reflect the two metacognition processes. Effort investment is related to the monitoring processes, since the athlete reflects on how the team functioned during the competition in terms of effort, intensity, and commitment. On the other hand, the perceived outcome dimension is related to metacognitive control. Here, the athlete is faced with outcome items and he/she is forced to decide explicitly about the end result compared to prior expectations. This task demands active monitoring over information (i.e., control) rather than merely monitoring.

### Limitations, Future Directions, and Implications

In self-serving bias, cognitive processes may be impaired by one’s need to preserve self-esteem (Campbell and Sedikides, 1999). Thus, under some circumstances, an unreliable response may be produced to rationalize or justify a certain outcome. As the PPTSQ aims at capturing high-order cognitive processes, the usefulness of the responses being provided in this study might be questioned. However, the reliability and validity of the PPTSQ provide evidence that professional and collegiate athletes can truly report on their team’s cognitive processes, possibly because reaching higher levels of performance requires strong abilities of analyzing cognitive processes (Breivik, 2013). To fully capture the utility of a subjective performance inventory, future PPTSQ studies must incorporate an inclusive approach that considers samples from novel or moderate skill levels as well as youth populations. Furthermore, future research may include additional performance measures (e.g., GPS and video analyses) alongside the PPTSQ and the outcome score. This line of investigation is especially prominent for validating the PPTSQ for a single match or a specific tournament or league.

From a methodological standpoint, the dataset was mostly based on Israeli participants, limiting the generalizability of the results. While the Israeli culture is mostly western, the use of the PPTSQ should be examined in other western cultures, such as in the US and western Europe, as well as in Asian, Eastern European, African, and other cultures. From a linguistic standpoint, although the common back-translation procedure (Brislin, 1986) was employed to accurately translate the PPTSQ, the questionnaire being directly validated in this study is the Hebrew version of the PPTSQ. Consequently, the original, English version of the PPTSQ should be examined. Such an examination can be conducted in future research.

Future directions may address the need for a valid and reliable measure for individual sports. As individual sports differ significantly from team sports in various psychological aspects (e.g., anxiety level), such a measure can facilitate the understating of one’s experience as he/she performs individually. Moreover, this measure can be used to better comprehend the relationship between psychological variables and individual performance.

Despite the prevalence of teams in the exercise and sport contexts, there is relatively little research involving teams’ mechanisms compared with other psychological topics in our field (Eys et al., 2019). Thus, the development of a validated tool that assesses the performance appraisals of sports teams intertwines the emotions and the pressures that exist in the competitive sport environment. These findings support the notion that addressing sport subjective performance inventory uncovers some blind spots in the understanding of team performance. Therefore, the literature of sport and exercise psychology can enrich its competencies. For instance, sport psychology scholars and practitioners can operationalize subjective team performance knowledge via the PPTSQ. Likewise, the completion of post-performance inventory can tailor the delivery of mental sessions following sport events and facilitate the preparation for upcoming competitions.

The development of the PPTSQ and the importance of perceived performance measure alongside an objective one supports an applied line of research on the importance of resourcefulness (e.g., Kennett, 1994). Thus, resourcefulness skills can enrich one’s adaptive abilities (i.e., tailoring appropriate solutions and utilizing self-management techniques) with minimal to no expenses (Goff, 2011). In cases of resourcefulness, the team can overcome barriers and exceed expectations (i.e., positive subjective performance evaluation) even if other, more resourced teams, do objectively better (e.g., are ranked higher).

Metacognition can facilitate team information processing in distinguishing good from poor information (Hinsz, 2004). In the long run, group-level metacognitive deliberation, as offered by the PPTSQ, can benefit team performance, as players and practitioners can learn from experience and evolve from successes and failures. The positive correlation between PPTSQ and TADI scores reflects this idea. Yet, further effort must establish a causal relationship between the two constructs.

## Conclusion

To conclude, the current study aimed at developing a validated tool of post-performance team members’ appraisals, namely, the PPTSQ. Objective measures of performance may overlook fundamental psychological mechanisms; hence, an integration of such a subjective inventory may provide valuable information in terms of interpreting the notion *team performance*. The content of the PPTSQ was driven by concepts from groups’ goal achievement in team sports (Brawley et al., 1992) and endorsed by two respected sport psychology scholars (i.e., content validity). The construct validity of the modified PPTSQ has been established in this study including two scales (i.e., effort investment and perceived outcome) with a demonstration of high reliability. Furthermore, the analyses confirmed the concurrent validity between PPTSQ factors and TADI and GEQ scores and the predictive validity between PPTSQ factors and objective measures of performance. Future research should address cultural, skill-level, and linguistic-related limitations in this study. As a valid and reliable performance measure that provides a more accurate understanding of the athletes’ competitive experience than the objective outcome measure commonly used, the PPTSQ has a great potential to contribute to sport psychology research. Moreover, the value of intervention programs, being tailored based on the PPTSQ’s results, must be investigated for the benefit of team performance.

## Data Availability Statement

The raw data supporting the conclusions of this article will be made available by the authors, without undue reservation.

## Ethics Statement

The studies involving human participants were reviewed and approved by the Florida State University IRB Committee. The patients/participants provided their written informed consent to participate in this study.

## Author Contributions

LG designed the study, collected and analyzed the data, and wrote the manuscript. AB helped with data collection and the write-up of the manuscript. TS analyzed the data and contributed to the write-up of the manuscript. GT supervised the project, contributed to the design of the study, data collection and analysis, and the write-up of the manuscript. All authors actively contributed to the writing process of the manuscript.

## Conflict of Interest

The authors declare that the research was conducted in the absence of any commercial or financial relationships that could be construed as a potential conflict of interest.

## Footnotes

1 There is no universally accepted guideline for acceptable or adequate omega reliability (nor to alpha; Lance et al., 2006). The ω coefficient should meet the same standards as alpha coefficients and a minimum of 0.75 would be preferred although this is merely a subjective guideline as Reise et al. (2013) suggested.

## References

[B1] Bar-Hillel M. (1980). The base-rate fallacy in probability judgments. *Acta Psychol.* 44 211–233. 10.1016/0001-6918(80)90046-3

[B2] Bass B. M. (1962). *The Orientation Inventory.* Palo Alto, CA: Consulting Psychologists Press.

[B3] BBC Sport (2018). *Catch-up**: Brazil v Switzerland.* Available online at: https://www.bbc.com/sport/live/football/43974754 (accessed September 6, 2020).

[B4] Brawley L. R. Carron A. V. Widmeyer W. N. (1992). The nature of group goals in sport teams: a phenomenological analysis. *Sport Psychol.* 6 323–333. 10.1123/tsp.6.4.323

[B5] Breivik G. (2013). Zombie-like or superconscious? a phenomenological and conceptual analysis of consciousness in elite sport. *J. Philos. Sport* 40 85–106. 10.1080/00948705.2012.725890

[B6] Brislin R. W. (1986). “The wording of translation of research instruments,” in *Field Methods in Cross-Cultural Research*, eds Lonner W. J. Berry J. W. (Beverly Hills, CA: Sage), 137–164

[B7] Campbell W. K. Sedikides C. (1999). Self-threat magnifies the self-serving bias: a meta-analytic integration. *Rev. Gen. Psychol.* 3 23–43. 10.1037/1089-2680.3.1.23

[B8] Cannon-Bowers J.A. Salas E. Converse S.A. (1993). “Shared mental models in expert team decision making,” in *Individual and Group Decision Making*, ed Castellan N.J. (Hillsdale, NJ: Lawrence Erlbaum Associates), 221–246.

[B9] Carron A. V. (1984). “Cohesion in sport teams,” in *Psychological Foundations of Sport*, eds Silva J. M. & Weinberg R. S. (Champaign, IL, Human Kinetics).

[B10] Carron A. V. Colman M. M. Wheeler J. Stevens D. (2002). Cohesion and performance in sport: a meta-analysis. *J. Sport Exerc. Psychol.* 24 168–188. 10.1123/jsep.24.2.168

[B11] Carron A. V. Widmeyer W. N. Brawley L. R. (1985). The development of an instrument to assess cohesion in sport teams: the group environment questionnaire. *J. Sport Psychol.* 7 244–266. 10.1123/jsp.7.3.244

[B12] Chatterji M. Sentovich C. Ferron J. Rendina-Gobioff G. (2002). Using an iterative model to conceptualize, pilot-test, and validate a teacher measure of reform readiness. *Educ. Psychol. Meas.* 62 444–465. 10.1177/001316402128774905

[B13] Chow G. M. Luzzeri M. (2019). Post-event reflection: a tool to facilitate self-awareness, self-monitoring, and self-regulation in athletes. *J. Sport Psychol. Action* 10 106–118. 10.1080/21520704.2018.1555565

[B14] Craft L. L. Magyar T. M. Bechker B. J. Feltz D. L. (2003). The relationship between the competitive state anxiety inventory-2 and sport performance: a meta-analysis. *J. Sport Exerc. Psychol.* 26 44–65. 10.1123/jsep.25.1.44

[B15] Duda J. L. (1998). *Advances in Sport and Exercise Psychology Measurement.* Morgantown: Fitness Information Technology.

[B16] Eccles D. W. Tenenbaum G. (2007). “A social-cognitive perspective on team functioning in sport,” in *Handbook of Sport Psychology* 3rd ed eds Tenenbaum G. & Eklund R. (New York: Wiley), 264–283. 10.1002/9781118270011.ch12

[B17] Eccles D. W. Tenenbaum G. (2004). Why an expert team is more than a team of experts: a social-cognitive conceptualization of team coordination and communication in sport. *J. Sport Exerc. Psychol.* 26 542–560. 10.1123/jsep.26.4.542

[B18] ESPN (2020). *Spanish Primera División Table 2019-20.* Available online at: https://www.espn.com/soccer/standings/_/league/ESP.1/season/2019 (accessed September 6, 2020).

[B19] Eys M. Bruner M. W. Martin L. J. (2019). The dynamic group environment in sport and exercise. *Psychol. Sport Exerc.* 42 40–47. 10.1016/j.psychsport.2018.11.001

[B20] Feltz D. L. Chow G. M. Hepler T. J. (2008). Path analysis of self-efficacy and diving performance revisited. *J. Sport Exerc. Psychol.* 30 401–411. 10.1123/jsep.30.3.401 18648112

[B21] Festinger L. Schachter S. Back K. (1963). *Social Pressures in Informal Groups.* Stanford, CA: Stanford University Press.

[B22] Filho E. S. M. Dobersek U. Gershgoren L. Becker B. Tenenbaum G. (2014). The cohesion-performance relationship in sport: A 10-year retrospective meta analysis. *Sport Sci. Health* 10 165–177. 10.1007/s11332-014-0188-7

[B23] Flavell J. H. (1979). Metacognition and cognitive monitoring: a new area of cognitive–developmental inquiry. *Am. Psychol.* 34 906–911. 10.1037/0003-066x.34.10.906

[B24] Gershgoren L. Filho E. M. Tenenbaum G. Schinke R. J. (2013). Coaching shared mental models in soccer: a longitudinal case study. *J. Clin. Sport Psychol.* 7 293–312. 10.1123/jcsp.7.4.293

[B25] Gershgoren L. Tenenbaum G. Gershgoren A. Eklund R. C. (2011). The effect of parental feedback on young athletes’ perceived motivational climate, goal involvement, goal orientation, and performance. *Psychol. Sport Exerc.* 12 481–489. 10.1016/j.psychsport.2011.05.003

[B26] Goff A. M. (2011). Stressors, academic performance, and learned resourcefulness in baccalaureate nursing students. *IntL. J. Nurs. Educ. Scholarsh.* 8:1. 10.2202/1548-923X.2114 21291410

[B27] Gould D. Guinan D. Greenleaf C. Medbery R. Peterson K. (1999). Factors affecting Olympic performance: perceptions of athletes and coaches from more and less successful teams. *Sport Psychol.* 13 371–394. 10.1123/tsp.13.4.371

[B28] Graham J. M. (2006). Congeneric and (essentially) tau-equivalent estimates of score reliability: What they are and how to use them. *Educ. Psychol. Meas.* 66 930–944. 10.1177/0013164406288165

[B29] Grand R. R. Carron A. V. (1982). “Development of a team climate questionnaire,” in *Psychology of Sport and Motor Behavior: Research and Practice*, eds Wankel L. M. & Wilberg R. B. (Alberta: University of Alberta).

[B30] Green S. B. Lissitz R. W. Mulaik S. A. (1977). Limitations of coefficient alpha as an index of test unidimensionality. *Educ. Psychol. Meas.* 37 827–838 10.1177/001316447703700403

[B31] Hancock G. R. An J. (2020). A closed-form alternative for estimating ω reliability under unidimensionality. *Meas. Interdiscip. Res. Perspect.* 18 1–14. 10.1080/15366367.2019.1656049

[B32] Hayes A. F. Coutts J. J. (2020). Use omega rather than Cronbach’s alpha for estimating reliability. but. *Commun. Methods Meas.* 14 1–24. 10.1080/19312458.2020.1718629

[B33] Hinkin T. R. (1995). A review of scale development practices in the study of organizations. *J. Manag.* 21 967–988. 10.1177/014920639502100509

[B34] Hinsz V. B. (2004). “Metacognition and mental models in groups: an illustration with metamemory of group recognition memory,” in *Team cogntion: Understanding the factors that drive process and performance* eds Salas E. & Fiore S. M. (Washington, DC: American Psychological Association), 33–58. 10.1037/10690-003

[B35] Johnson T. E. Lee Y. Lee M. O’Connor D. L. Khalil M. K. Huang X. (2007). Measuring sharedness of team-related knowledge: design and validation of a shared mental model instrument. *Hum. Resour. Dev. Intl.* 10 437–454. 10.1080/13678860701723802

[B36] Kahneman D. Tversky A. (1973). On the psychology of prediction. *Psychol. Rev.* 80 237–251.

[B37] Kennett D. J. (1994). Academic self-management counselling: preliminary evidence for the importance of learned resourcefulness on program success. *Stud. High. Educ.* 19 295–307. 10.1080/03075079412331381890

[B38] Kline R. B. (2015). *Principles and Practice of Structural Equation Modeling* 4th ed New York: The Guilford Press

[B39] Lance C. E. Butts M. M. Michels L. C. (2006). The sources of four commonly reported cutoff criteria: what did they really say? *Organ. Res. Methods* 9 202–220. 10.1177/1094428105284919

[B40] Lazarus R. S. (2000). How emotions influence performance in competitive sport. *Sport Psychol.* 14 229–252. 10.1123/tsp.14.3.229

[B41] Li F. Harmer P. (1996). Confirmatory factor analysis of the group environment questionnaire with an intercollegiate sample. *J. Sport Exerc. Psychol.* 18 49–63. 10.1123/jsep.18.1.49

[B42] Martens R. Landers D. M. Loy J. W. (1971). *Sport Cohesiveness Questionnaire.* Champaign, IL: University of Illinois.

[B43] McDonald R. P. (1999). *Test Theory: A unified Approach.* Mahwah, NJ: Erlbaum.

[B44] Medvec V. H. Madey S. F. Gilovich T. (1995). When less is more: counterfactual thinking and satisfaction among Olympic medalists. *J. Pers. Soc. Psychol.* 69 603–610. 10.1037/0022-3514.69.4.603 7473022

[B45] Micklewright D. Papadopoulou E. Parry D. Hew-Butler T. Tam N. Noakes T. (2009). Perceived exertion influences pacing among ultramarathon runners but post-race mood change is associated with performance expectancy. *South Afr. J. Sports Med.* 21 167–172.

[B46] Moritz S. E. Feltz D. L. Fahrbach K. R. Mack D. E. (2000). The relation of self-efficacy measures to sport performance: a meta-analytic review. *Res. Q. Exerc. Sport* 71 280–294. 10.1080/02701367.2000.10608908 10999265

[B47] Nelson T. Narens L. (1990). Metamemory: a theoretical framework and new findings. *Psychol. Learn. Motiv.* 26 125–173. 10.1016/s0079-7421(08)60053-5

[B48] Rasker P. C. Post W. M. Schraagen J. M. C. (2000). Effects of two types of intra-team feedback on developing a shared mental model in command and control teams. *Ergonomics* 43 1167–1189. 10.1080/00140130050084932 10975179

[B49] Reise S. P. Bonifay W. E. Haviland M. G. (2013). Scoring and modeling psychological measures in the presence of multidimensionality. *J. Pers. Assess.* 95 129–140. 10.1080/00223891.2012.725437 23030794

[B50] Tenenbaum G. (2003). “Expert athletes: an integrated approach to decision making,” in *Expert Performance in Sports* eds Starkes J.L. & Ericsson K.A. (Champaign, IL: Human Kinetics), 191–218

[B51] Tenenbaum G. E. Eklund R. C. Kamata A. E. (2012). *Measurement in Sport and Exercise Psychology.* Champaign, IL: Human Kinetics.

[B52] Tenenbaum G. Gershgoren L. (2014). “Decision making,” in *Encyclopedia of Sport & Exercise Psychology*, Vol. 4, eds Eklund R. Tenenbaum G. (Thousand Oaks, CA: SAGE Publications, Inc.), 190–192.

[B53] Transfermarket (2020a). *FC Barcelona Transfer Record 2019–20.* Available online at: https://www.transfermarkt.com/fc-barcelona/transfers/verein/131/saison_id/2019 (accessed September 6, 2020).

[B54] Transfermarket (2020b). *Granada Cf Historical Rankings.* Available online at: https://www.transfermarkt.com/fc-granada/platzierungen/verein/16795 (accessed September 6, 2020).

[B55] Trizano-Hermosilla I. Alvarado J. M. (2016). Best alternatives to Cronbach’s alpha reliability in realistic conditions: congeneric and asymmetrical measurements. *Front. Psychol.* 7:769. 10.3389/fpsyg.2016.00769 PMC4880791 27303333

[B56] Vaughn B. K. Daniel S. R. (2012). “Conceptualizing validity,” in *Measurement in Sport and Exercise Psychology* eds Tenenbaum G. Eklund R. C. Kamata A. (Champaign, IL: Human Kinetics), 33–39

[B57] Webber S. S. Chen G. Payne S. C. Marsh S. M. Zaccaro S. J. (2000). Enhancing team mental model measurement with performance appraisal practices. *Organ. Res. Methods* 3 307–322. 10.1177/109442810034001

[B58] Weiner B. (Ed.). (1974). *Achievement Motivation and Attribution Theory.* New York: General Learning Press.?

[B59] Widmeyer W. N. Brawley L. R. Carron A. V. (1985). *The Measurement Of Cohesion in Sport Teams: the Group Environment Questionnaire.* London, CA: Sports Dynamics. 2924223

