# Supplementary material for: Does the League Table Lie? The Development and Validation of the Perceived Performance in Team Sports Questionnaire (PPTSQ)
Source: Front Psychol. 2021 Jan 21;11:615018. doi: 10.3389/fpsyg.2020.615018 (PMC7859492; doi:10.3389/fpsyg.2020.615018)
Supplement: Supplementary file 1 [file Data_Sheet_1.docx]

**Appendix A – The Original PPTSQ**

**During this competitive season/tournament, my team:**

| Item # |  |  |  |  |  |
| --- | --- | --- | --- | --- | --- |
| 1 | Exhibited very low game quality |  | Exhibited moderate game quality |  | Exhibited very high game quality |
|  | 1 | 2 | 3 | 4 | 5 |
| 2 | Fell short to meet its  pre-season expectations |  | Exactly reached its pre-season expectations |  | Exceeded its pre-season expectations |
|  | 1 | 2 | 3 | 4 | 5 |
| 3 | Was far from reaching its full potential in competitions |  | Partially reached its potential in competitions |  | Fully reached its potential in competitions |
|  | 1 | 2 | 3 | 4 | 5 |
| 4 | Played with very low intensity |  | Played with moderate intensity |  | Played with very high intensity |
|  | 1 | 2 | 3 | 4 | 5 |
| 5 | Did not achieve any of its collective goals |  | Partially achieved its collective goals |  | Fully achieved its collective goals |
|  | 1 | 2 | 3 | 4 | 5 |
| 6 | Performed much worse than expected |  | Performed as expected |  | Performed much better than expected |
|  | 1 | 2 | 3 | 4 | 5 |
| 7 | Was ranked much lower than initially expected |  | Was ranked as initially expected |  | Was ranked much higher than initially expected |
|  | 1 | 2 | 3 | 4 | 5 |
| 8 | Exerted very low effort in games |  | Exerted reasonable effort in games |  | Exerted very high effort in games |
|  | 1 | 2 | 3 | 4 | 5 |
| 9 | Implemented its strategies ineffectively |  | Implemented its strategies at a fairly effective level |  | Implemented its strategies extremely effectively |
|  | 1 | 2 | 3 | 4 | 5 |
| 10 | Failed to reach its objectives |  | Reached its objectives |  | Reached its objectives above expectations |
|  | 1 | 2 | 3 | 4 | 5 |
| 11 | Played with a low commitment level |  | Played with moderate commitment levels |  | Played with a very high commitment level |
|  | 1 | 2 | 3 | 4 | 5 |
| 12 | Played terribly within the team’s system/formation |  | Played reasonably within the team’s system/formation |  | Played perfectly within the team’s system/formation |
|  | 1 | 2 | 3 | 4 | 5 |

*Effort investment* scale: Items 4, 8, & 11; *Skills Execution* scale: Items 1, 3, 6, 9, & 12; *Perceived outcome* scale: Items 2, 5, 7, & 10.

**Appendix B – The Final PPTSQ**

Please read the following statements, which pertain to your team’s performance during the last season/tournament, and rank each on a scale ranging from “1” (*strongly disagree*) to “5” (*strongly agree*). Please consider your team as a collective unit.

**During this competitive season/tournament, my team:**

| Item # |  |  |  |  |  |
| --- | --- | --- | --- | --- | --- |
| 1 | Fell short to meet its  pre-season expectations |  | Exactly reached its pre-season expectations |  | Exceeded its pre-season expectations |
|  | 1 | 2 | 3 | 4 | 5 |
| 2 | Played with very low intensity |  | Played with moderate intensity |  | Played with very high intensity |
|  | 1 | 2 | 3 | 4 | 5 |
| 3 | Was ranked much lower than initially expected |  | Was ranked as initially expected |  | Was ranked much higher than initially expected |
|  | 1 | 2 | 3 | 4 | 5 |
| 4 | Exerted very low effort in games |  | Exerted reasonable effort in games |  | Exerted very high effort in games |
|  | 1 | 2 | 3 | 4 | 5 |
| 5 | Failed to reach its objectives |  | Reached its objectives |  | Reached its objectives above expectations |
|  | 1 | 2 | 3 | 4 | 5 |
| 6 | Played with a low commitment level |  | Played with moderate commitment levels |  | Played with a very high commitment level |
|  | 1 | 2 | 3 | 4 | 5 |

*Effort Exertion* scale: Items 2, 4, & 6; *Perceived Outcome* scale: Items 1, 3, & 5
